# Radioprotective Effect of Melatonin on Radiation-Induced Lung Injury and Lipid Peroxidation in Rats

**DOI:** 10.22074/cellj.2015.517

**Published:** 2015-04-08

**Authors:** Raziyeh Tahamtan, Ali Shabestani Monfared, Yasser Tahamtani, Alireza Tavassoli, Maasoomeh Akmali, Mohammad Amin Mosleh-Shirazi, Mohammad Mehdi Naghizadeh, Danial Ghasemi, Mojtaba Keshavarz, Gholam Hassan Haddadi

**Affiliations:** 1 Department of Biophysics and Biochemistry, Cellular and Molecular Biology Research Centre, Babol University of Medical Sciences, Babol, Iran; 2 Department of Stem Cells and Developmental Biology at Cell Science Research Center, Royan Institute for Stem Cell Biology and Technology, ACECR, Tehran, Iran; 3 Department of Pathology, Fasa University of Medical Sciences, Fasa, Iran; 4 Department of Biochemistry, Faculty of Medicine, Shiraz University of Medical Sciences, Shiraz, Iran; 5 Department of Radiotherapy, Physics Unit, Namazi Hospital, Shiraz University of Medical Sciences, Shiraz, Iran; 6 Department of Biostatistics, Fasa University of Medical Sciences, Fasa, Iran; 7 Student Research Committee, Shiraz University of Medical Sciences, Shiraz, Iran; 8 Young Researchers and Elite Club, Shiraz Branch, Islamic Azad University of Shiraz, Shiraz Iran; 9 Department of Medical Physics, Fasa University of Medical Sciences, Fasa, Iran

**Keywords:** Radiation, Melatonin, Pulmonary Fibrosis

## Abstract

**Objective:**

Free radicals generated by ionizing radiation attack various cellular components such as lipids. The lung is a very radiosensitive organ and its damage is a doselimiting factor in radiotherapy treatments. Melatonin (MLT), the major product of the pineal gland acts as a radioprotective agent. This study aims to investigate the radioprotective effects of MLT on malondialdehyde (MDA) levels and histopathological changes in irradiated lungs.

**Materials and Methods:**

In this experimental study, a total of 62 rats were divided into five groups. Group 1 received no MLT and radiation (unT), group 2 received oral MLT (oM), group 3 received oral MLT and their thoracic areas were irradiated with 18 Gy (oMR), group 4 received MLT by intraperitoneal (i.p.) injection and their thoracic areas were irradiated with 18 Gy (ipM-R), group 5 received only 18 Gy radiation in the thoracic area (R). Following radiotherapy, half of the animals in each group were sacrificed at 48 hours for evaluation of lipid peroxidation and early phase lung injuries. Other animals were sacrificed in the eighth week of the experiment for evaluation of the presence of late phase radiation induced lung injuries.

**Results:**

Pre-treatment of rats with either i.p injection (p<0.05) and oral administration of MLT (p<0.001) significantly reduced MDA levels in red blood cell (RBC) samples compared to the R group. Furthermore, i.p. injection of MLT decreased MDA levels in plasma and tissue (p<0.05). In the early phase of lung injury, both administration of MLT significantly increased lymphocyte (p<0.05) and macrophage frequency (p<0.001). MLT reduced the lung injury index in the lungs compared to the R group (p<0.05).

**Conclusion:**

The result of this study confirms the radioprotective effect of MLT on lipid peroxidation, and in both early and late phases of radiation induced lung injuries in an animal model.

## Introduction

Radiotherapy is one of the most common, important techniques for cancer treatment which is performed with the intent to cure or for palliation (1,2). The lungs are radiosensitive organs. In patients with thorax and chest wall malignancies that include the breasts, lungs, esophagus, lymphomas or any other mediastinal neoplasms, irradiation of the lungs is inevitable (3,4). The radiation dose and irradiated volume are limiting factors in chest radiotherapy and should be taken into consideration for prevention of lung injuries (4,6). Lung injuries are divided into two distinct phases. The first or early phase is called radiation pneumonitis ( acute syndrome ) as evidenced by alveolar edema, alveolar neutrophils, alveolar erythrocytes, and foamy macrophages according to histopathological evaluation. These injuries appear at approximately 1-6 months after radiation therapy in 10-15% of patients who underwent whole lung irradiation (7,9). According to studies of histopathological changes in this phase, extensive alveolar damage is considered as the first symptom of a lung injury (10). The second or latent phase ( chronic syndrome ) is the occurrence of pulmonary fibrosis months to years after radiotherapy. Vascular injury in the early phase of an inflammatory response is one of the prominent symptoms which progresses with extensive increase in collagen and extracellular matrix, and finally leads to tissue fibrosis (9). 

However, ionizing radiation can damage living cells due to free radical generation during water radiolysis in the cells (11). Three of the most reactive species generated in water radiolysis are the aqueous electron ( eaq ), hydrogen radical ( H° ) and hydroxyl radical ( OH° ). OH° is the most damaging free radical in cells (12). Proteins, carbohydrates, lipids, and also DNA are critical molecules susceptible to damage by free radicals (13). Since the cell membrane consists of high amounts of lipids, lipid peroxidation is considered to be an essential reason for cell membrane destruction and one of the main factors involved in tissue damage by oxygen free radicals (14). The final product of lipid peroxidation is malondialdehyde ( MDA ) which is highly cytotoxic and prevents the action of antioxidant enzymes (15,17). 

It has been demonstrated that some chemical agents ( radioprotectors ) protect cells against radiation-induced normal tissue injuries (18). Radioprotectors should be delivered at the time of radiation therapy at an adequate concentration to overcome oxidative stress (7). Protective properties of these agents rely on scavenging free radicals generated by radiation and antioxidant activity (19). Cysteine and cysteamine, as sulfhydryl compounds have free radical scavenging action and are the first compounds known as radioprotectors (20). However, the adverse effects of these compounds ( e.g., amifostine ) limit their use at the required protective doses (1). Melatonin ( MLT; N-acetyl-5-methoxytryptamine ) is an endogenous hormone synthesized by the pineal gland in vertebrates with a half-life of 30 to 57 minutes in serum (21). Recently it has been demonstrated that MLT has radioprotective properties and in the rat this protective effect has been observed at doses of 100-200 mg/ kg (14,22). The molecular mechanism of its radioprotection is due to the direct scavenging of OH° (7,23,24), therefore MLT can reduce the risk of OH° damage to nearby critical molecules (13,25). The high lipid solubility of MLT enables it to pass through the cell membrane and all morphophysiological barriers, preventing the initiation of events that result in lipid peroxidation (26). In addition to its direct action MLT can enhance the activity of antioxidant enzymes such as superoxide dismutase ( SOD ) or glutathione peroxidase ( GSH ) (14,27,28). In addition, MLT’s metabolites -N1-acetyl-N2-formyl-5-methoxykynuramine ( AFMK ) and N-acetyl-5-methoxykynuramine ( AMK ) are known as free radical scavengers (29). The present study focuses on the effectiveness of MLT as a radioprotector reagent using oral administration and intraperitoneal ( i.p. ) injection after localized irradiation of the lungs. Additionally, the effects of MLT on acute and chronic lung injuries have been evaluated by histopathological analyses at two time points after lung irradiation. Considering radiation’s effect on lipids, we determined the MDA levels in lung tissue and blood samples. 

## Materials and Methods

### Experimental design

In this experimental study, a total of 62 male, 10-to-12-week-old Wistar rats of approximately 250-300 g in weight were purchased from Shiraz University Laboratory and kept according to the Guidelines for the Care and Use of Laboratory Animals as adopted by the Ethics Committee of Shiraz University of Medical Sciences. All rats were acclimatized for a minimum of ten days before the initiation of the experiments. Then, rats were allocated into the following five groups: I. the first group ( unT ) received no MLT or radiotherapy, II. the second group ( oM group ) received MLT orally and underwent sham radiotherapy, III. the third experimental group ( oM-R group ) received oral MLT and after 30 minutes underwent radiation, IV. the fourth group received an i.p. injection of MLT and after 30 minutes underwent radiation ( ipM-R group ) and V. the fifth group ( R group ) received no MLT but underwent radiation therapy. 

### Melatonin

MLT ( off-white solid, Enzolife Science Corporation, UK ) was prepared at a concentration of 1% with by dissolution in ethanol ( Sigma, USA ) and dilution in 0.9% sodium chloride ( Shimiran, Iran ). The administration dose of MLT was 100 mg/kg according to previous reports (11,30,31). MLT was administered orally in the oM and oM-R groups and injected i.p. in the ipM-R group 30 minutes prior to radiation therapy or sham radiation ( in the oM group ). In the unT groups, 0.9% sodium chloride was prepared at the same volume as MLT and orally administered. 

### Radiotherapy

Prior to whole thorax irradiation, we anesthetized the animals with i.p. injections of ketamine ( Alphasan, Netherland Bv ) at doses of 60-90 mg/kg and xylazine ( Alfasan, Woerden, Holland ) at doses of 6-10 mg/kg. Then, the animals were placed on a Plexiglas tray in the supine position by taping their extremities. We chose an 18 Gy single dose x-radiation according to previous studies (30,32). Rats in the oM-R, ipMR and R groups were irradiated with a 6 MV X-ray linear accelerator machine ( Elekta Compact 6 MV, China ) from a source-to-surface distance of 100 cm. A single dose of 18 Gy x-radiation was delivered to the whole thorax area at a dose rate of 350 monitor unit ( MU ). To increase lung dose to the maximum, a bolus with a 1 cm thickness was placed at a distance of 1 cm above the thorax. The dose was calculated for the central axis at a depth of 3 cm. For the rats in the unT and oM groups, sham radiation therapy was delivered over the same fraction duration. 

### Sample preparation

At 48 hours after irradiation of the lungs, half of the animals in each group were anesthetized with ether. Then, 3-5 ml blood samples were collected through an intra-cardiac withdrawal and centrifuged at 1200 g for 15 minutes to separate plasma and erythrocytes. These procedures were performed at a temperature of 4˚C. Red blood cells ( RBC ) were washed and susn pended three times in isotonic solution ( 1/3 volume ). The plasma and RBC suspensions were maintained at -20˚C. We dissected the rats’ lungs and homogenized the right lung from each animal was in cold phosphate buffer ( Sigma, USA, 1/5 weight/volume ) on ice for 30 seconds. The homogenate was centrifuged at 1500 g for 10 minutes at 4˚C. The supernatant was stored at -20˚C for biochemical measurements. Left lungs were kept in 10% buffered formaldehyde ( Shimiran, Iran ) for histopathological analyses. The remaining rats from each group were sacrificed eight weeks following radiation therapy or sham radiation. Their lungs were dissected out and instilled with 10% buffered formaldehyde. To prepare histopathological slides, the lung samples from either 48 hours or 8 weeks were embedded in paraffin ( Shimiran, Iran ). Then, samples were sliced into 5 μm thick sections and stained using hematoxylin and eosin ( H&E ) and Masson’s trichrome. 

### Biochemistry assay

The concentration of MDA as a product of lipid peroxidation in plasma, RBC and lung tissue homogenate were determined as previously described (33). Briefly, 200 µL of samples ( plasma, RBC, or tissue homogenate ) were incubated with 1 ml of 86% thiobarbituric acid ( TBA; Sigma, USA ) solution in 20% trichloroacetic acid ( Sigma, USA, 20 g trichloroacetic acid, 0.86 g TBA, 100 ml distilled water ) in a boiling water bath for 20 minutes. After another 20-minute ice bath, the absorbance was read at 532 nm against a blank TBA solution using a spectrophotometer ( Shimadzu, UV-1700, Japan ). The absorbance of supernatants was measured at 532 nm. The MDA levels were expressed as nmol/ml for plasma and RBC samples, and nmol/g of tissue for lung tissue homogenate. 

### Histopathological evaluation

Acute and chronic histopathological changes were evaluated under the light microscope ( Nikon, YS 100, Japan ) by personnel blinded to the samples. Semi-quantitative scoring of each variable was performed by a histopathologist using the following scale: 0 ( no change ), 1 ( slight ), 2 ( mild ), 3 ( moderate ), and 4 ( severe ) injury. The descriptive items for radiation-induced lung injuries were the presence of 114 neutrophils, RBCs ( presence of erythrocytes in the alveolar sac or perivascular region ), macrophages ( presence of transmigrated foamy macrophages in the alveolar sac ), lymphocytes ( presence of transmigrated lymphocytes in the alveolar sac or perivascular region ), incidence of edema ( presence of fluid in the alveolar sac ), hyaline arteriosclerosis ( presence of hyaline in the vascular wall ), alveolar fibrosis ( presence of collagen in the alveolar sac ), and collapse ( absence of air in the alveolar sac and presence of apposed epithelial cells in the alveolar wall ). We created a radiation-induced lung injury index by adding the scores of the eight previously mentioned items which included the presence of neutrophils, erythrocytes, transmigrated foamy macrophages, and lymphocytes in the alveolar sac or perivascular region and the incidence of edema, hyaline arteriosclerosis, alveolar fibrosis and collapse in the histopathological samples. 

### Statistical analysis

Biochemical data were statistically analyzed using one-way analysis of variance ( ANOVA ) followed by Tukey’s post hoc test. Histopathological evaluations were analyzed by the Chi-square test ( median test ) and a pair-wise comparison with the Mann-Whitney test. All data were presented as mean ± standard deviation ( SD ) and analyzed using statistical package for social sience ( SPSS 20; SPSS Inc., Chicago, IL, USA ). Significance was considered to be p<0.05. 

## Results

### Effects of MLT on MDA levels

**Fig.1:**
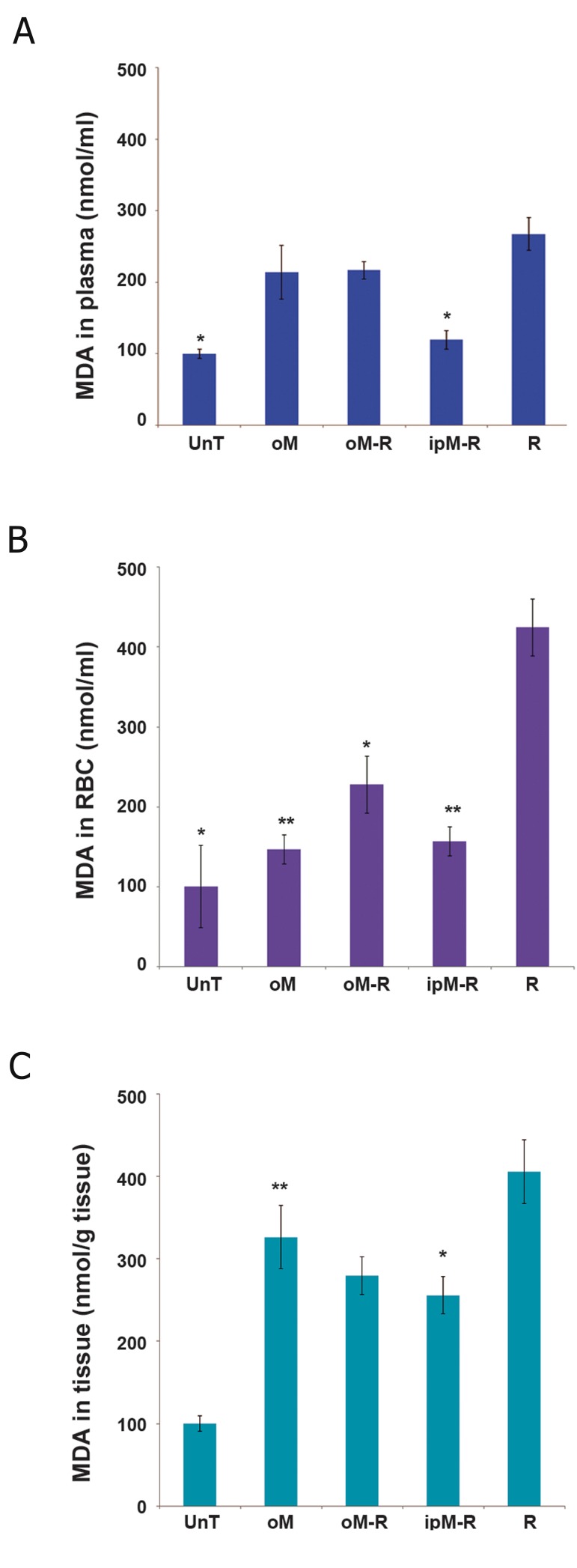
Effects of MLT on MDA levels in ( A ) plasma, ( B ) RBCs and ( C ) lung tissue after 48 hours in the five study groups. The MDA level of the R group was significantly higher than the unT group. Pre-treatment with oral MLT in the oM-R group significantly reduced MDA levels in RBC samples; i.p injection of MLT decreased MDA levels in plasma, RBCs and lung tissue in the ipM-R group. Data are mean ± SEM in the five study groups. *; P<0.05 and **; P<0.001 compared to the R group, MLT; Melatonin, MDA; Malondialdehyde, RBC; Red blood cells, unT; Untreated, oM; Oral MLT, oM-R; Oral MLT+radiation, ipM-R; i.p. injection of MLT+radiation, R; Radiation and SEM; Standard error of mean.

We measured MDA levels in rats’ plasma, RBCs and lung tissue homogenates in order to investigate the effects of MLT on radiation-induced lipid peroxidation. As shown in figure 1 there were significantly higher MDA levels in plasma, RBC (Fig.1A,B;p<0.05) and lung tissue (Fig.1C;p<0.001) in the R group compared to the unT group. There was a statistically significant decrease in plasma MDA levels in rats who received i.p. injections of MLT before radiation (Fig.1A;p<0.05). According to blood MDA levels, there were significant decreases in lipid peroxidation in the oM-R ( p<0.05 ) and ipM-R ( p<0.001 ) groups. The results showed that i.p injections of MLT were more effective in reducing MDA levels in RBCs than oral administration of MLT (Fig.1B). The results in lung tissue samples indicated a significant ( p<0.05 ) decrease in MDA level in the ipM-R group compared with the R group (Fig.1C). 

## Effects of MLT in the acute phase of radiationinduced lung injury

At 48 hours post-radiation therapy, acute changes in the rats’ lungs were histopathologically evaluated. Table 1 shows the mean ± S.D of acute changes in terms of the lung injury index for all experimental groups. The results showed significant increases in fibrosis symptoms, collapse incidence, and lymphocyte frequency in the R group ( 2.38 ± 1.22 ) compared with 0 in the unT group ( p<0.05 ). 

**Fig.2:**
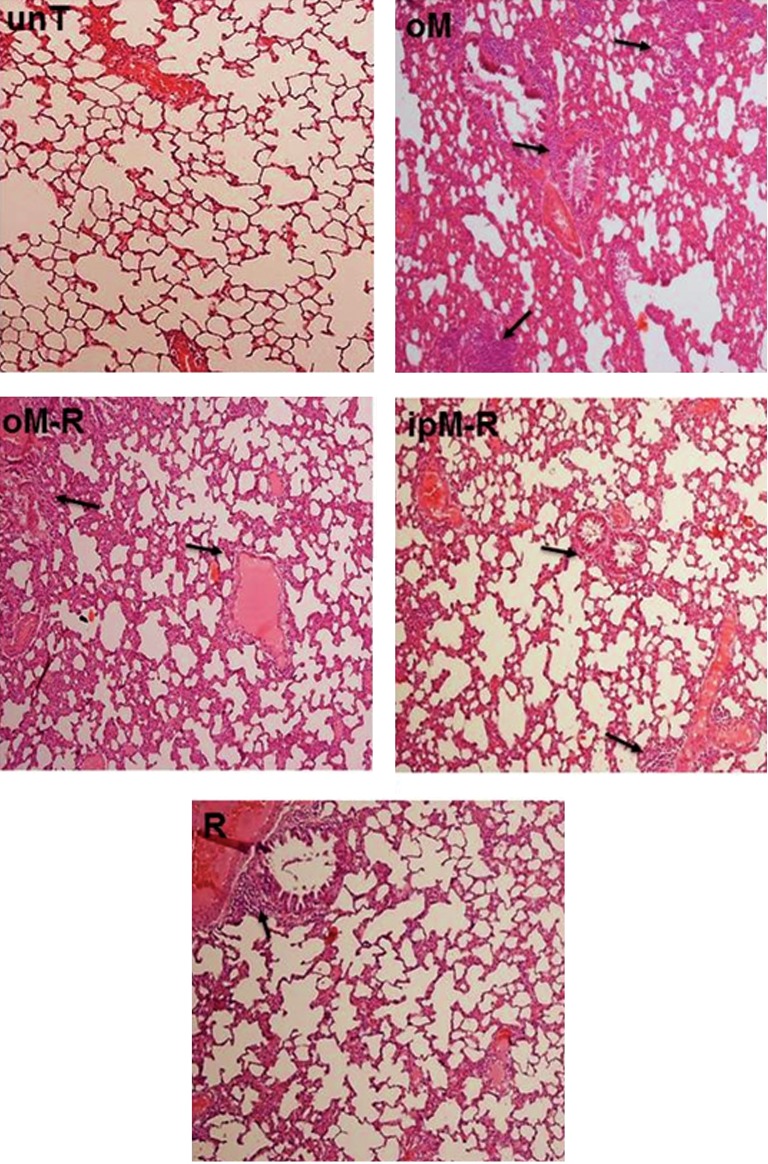
Histopatdological effects of MLT in the acute phase ( 48 hours ) of lung injury post 18 Gy irradiation. Increased numbers of lymphocytes and macrophages were observed in the oM, oM-R and ipM-R groups. Arrows indicate lymphocyte accumulation in lung tissue ( ×100 magnification, H&E staining ). MLT; Melatonin, unT; Untreated, oM; Oral MLT, oM-R; Oral MLT radiation, ipM-R; i.p. injection of MLT+radiation, R; Radiation group and H&E; Hematoxilin & eosin.

Although the collapse incidence significantly increased ( p<0.05 ) in the R group compared with the oM group, the frequency of lymphocytes, macrophages and hyaline arteriosclerosis were less in the R group compared to the oM group ( p<0.05 ). As shown in table 1, the lung injury index was higher in the oM-R ( 3.08 ± 1.24 ) and i.pM-R ( 3.07 ± 0.58 ) groups compared with the R group ( 2.38 ± 1.22 ). The higher index in the oM-R group was the result of significant increases in the numbers of lymphocytes ( p<0.05 ) and macrophages ( p<0.001 ). In contrast, oral administration of MLT before radiation significantly reduced the collapse incidence and frequency of RBC compared with the R group ( p<0.05 ). There were similar effects in reduction of RBCs, fibrosis and edema ( p<0.05 ) in the ipM-R group compared with the R group. The results showed increased numbers of macrophages ( p<0.001 ) and lymphocytes ( p<0.05 ) in the ipM-R group which resulted in a higher lung injury index in this group compared to the R group (Fig.2). 

**Table 1:** Radiation-induced lung injury index at 48 hours and 8 weeks post-radiation in the study groups


	unT	oM	oM-R	ipM-R	R	P value

**48 hours**	0	3.8 ± 0.75	3.08 ± 1.24	3.07 ± 0.53	2.38 ± 1.22	0.043
**8 weeks**	0	2.75 ± 0.87	6.75 ± 1.64	5.71 ± 1.19	10.7 ± 1.3	0.002


The values are written as mean ± SD. unT; Untreated, oM; Oral melatonin, oM-R; Oral MLT+radiation, ipM-R; i.p. injection of MLT+radiation, R; Radiation group and SD; Standard deviation.

**Fig.3:**
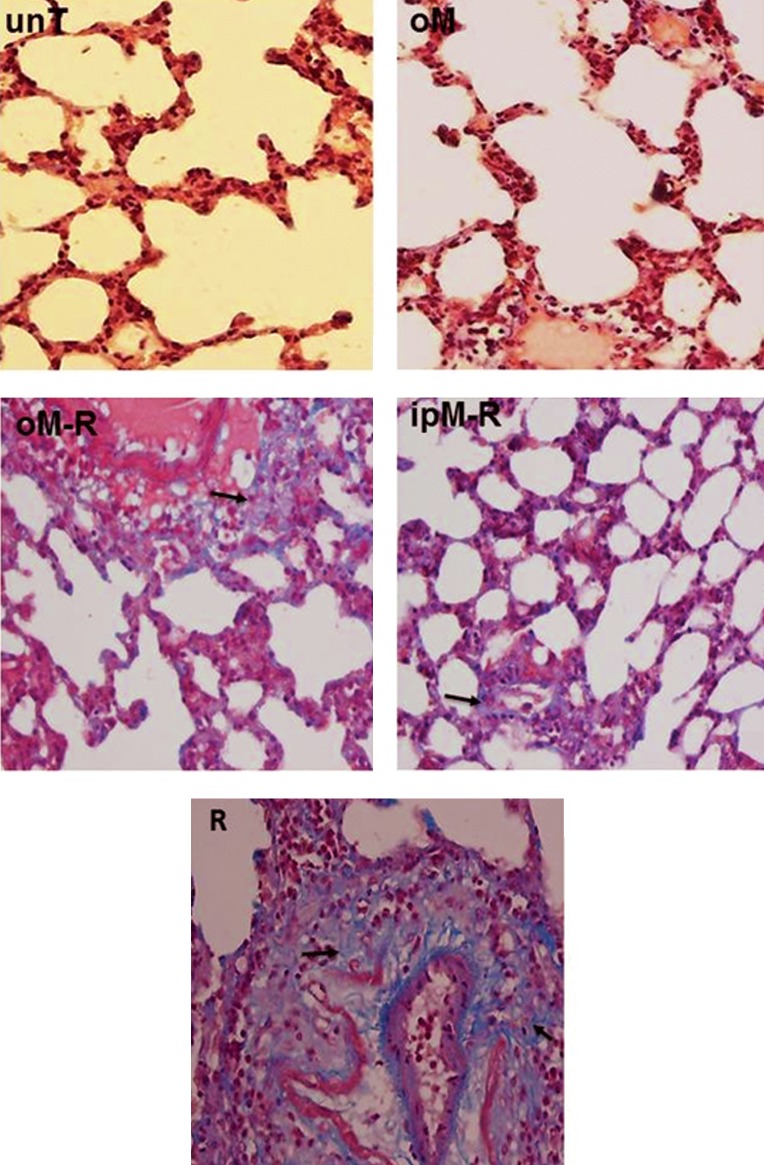
Histopathological effects of MLT in the chronic phase ( 8 weeks ) of lung injury post 18 Gy irradiation. Increased fibrosis is seen in the R group. Pre-treatment with MLT in the oM-R and ipM-R groups significantly decreased fibrosis. Arrows indicate collagen accumulation as a sign of fibrosis incidence in lung tissue ( ×400 magnification, Masson trichrome staining ). MLT; Melatonin, unT; Untreated;, oM; Oral MLT, oM-R; Oral MLT+radiation, ipM-R; i.p. injection of MLT+radiation and R; Radiation group.

## Discussion

In order to investigate the level of lipid peroxidation after radiation therapy, we determined MDA levels in RBCs and lung tissue (Fig.1). In accordance with previous reports (15, 34-36), our results indicated that lipid peroxidation increased in blood and tissues after localized irradiation of the lung tissue. The highest concentration of MLT was in the cell membrane (37), thus the present investigation focused on protective action of exogenous MLT against radiation-induced lipid peroxidation. Radioprotective action of MLT against lipid peroxidation was previously reported in various studies that used different doses of ionizing radiation and a wide doses range of MLT (14, 38-40). Our results showed that i.p. injection of MLT before radiotherapy decreased MDA levels in lung tissue. Its protective effect on irradiated tissue was in accordance with the results reported in a study by Undeger et al. (36). The levels of MDA in blood samples in the ipM-R group significantly decreased compared to the R group. This finding was in accordance with those by Kaya et al. (14). The effect of MLT on reduction of MDA levels related to its properties as an effective free radical scavenger (41).

In the current study, histopathological evaluation was performed during the acute phase of the lung injury (Table 1). There was an increase in the collapse incidence in the R group. This might be the result of damages incurred by type II cells in the lungs. These cells produce surfactant in the alveolar sacs. Injuries to type II cells lead to reductions in surfactant production. Therefore collapse occurs after surfactant reduction in alveolar sacs (42).

Radiation as a oxidative stress agent stimulates the immune response (43). Our obtained results have shown a significant increase in lymphocytes and macrophages in the R group. In addition, our results showed a gradual increase in collagen production after radiation which ultimately resulted in fibrosis (2). Since MLT plays an immunomodulatory role due to its binding sites in blood lymphocytes, it can influence the immune system by stimulating cytokine production (44, 45). As observed in this study, both forms of MLT (oral and i.p.) stimulated the immune response by producing lymphocytes and macrophages (Fig.2).

In the chronic phase of the lung injury, in accordance with previous *in vivo* studies (30, 46-49), our results showed a significant increase in the lung injury index in the R group (Table 1). Activation of the immune system and initiation of inflammation are responses to overcome injuries. Residual inflammation causes chronic inflammation. Considering molecular effects of MLT in the immune system, it can play a role in controlling symptoms of chronic inflammation (45). In the chronic phase of the lung injury our results indicated that administration of MLT, as either oral or i.p. injection, changed the lung injury index by decreasing factors associated with lung injuries (Table 1). According to our findings, i.p. injections of MLT were more efficient against radiation injuries. This might be related to the bioavailability of MLT after injection which was previously reported by Yeleswaram et al. (50). In summary, the obtained results demonstrated that MLT could down regulate cell injury by scavenging free radicals and stimulating antioxidant enzymes within cells.

## Conclusion

In this study, the results showed that either i.p. injections or oral administration of MLT showed biochemical and pathological benefits in radiationinduced normal lung tissue injuries in the rat model. The results of the present study could be a basis for further studies that attempt to reduce lung injuries in patients who have undergone thorax irradiation. However, further experiments are required to investigate the molecular mechanisms related to the radioprotective function of MLT in lungs. 
